# Identification of Global Alteration of Translational Regulation in Glioma *In Vivo*

**DOI:** 10.1371/journal.pone.0046965

**Published:** 2012-10-03

**Authors:** Karim Helmy, John Halliday, Elena Fomchenko, Manu Setty, Ken Pitter, Christoph Hafemeister, Eric C. Holland

**Affiliations:** 1 Department of Cancer Biology and Genetics, Memorial Sloan-Kettering Cancer Center, New York, New York, United States of America; 2 Brain Tumor Center, Memorial Sloan-Kettering Cancer Center, New York, New York, United States of America; 3 Department of Medicine, New Jersey Medical School, University of Medicine and Dentistry of New Jersey, Newark, New Jersey, United States of America; 4 Graduate School of Biomedical Sciences, University of Medicine and Dentistry of New Jersey, Newark, New Jersey, United States of America; 5 Gerstner Sloan-Kettering Graduate School of Biomedical Sciences, New York, New York, United States of America; 6 Weill Medical College of Cornell University, New York, New York, United States of America; 7 Computational Biology Center, Memorial Sloan-Kettering Cancer Center, New York, New York, United States of America; 8 Department of Biology, New York University, New York, New York, United States of America; 9 Departments of Neurosurgery, Neurology and Surgery, Memorial Sloan-Kettering Cancer Center, New York, New York, United States of America; University of Illinois College of Medicine, United States of America

## Abstract

Post-transcriptional regulation of gene expression contributes to the protein output of a cell, however, methods for measuring translational regulation in complex *in vivo* systems are lacking. Here, we describe a sensitive method for measuring translational regulation in defined cell populations from heterogeneous tissue *in vivo*. We adapted the translating ribosome affinity purification (TRAP) methodology to measure the relative occupancy of individual mRNA transcripts in translating ribosomes in the Olig2-positive tumor cell population in a genetically engineered mouse model (GEM) of glioma. Global measurement of paired ribosome-bound and total cellular mRNA populations from tumor cells *in vivo* identified a broad distribution of relative ribosome occupancies amongst mRNA species that was highly reproducible across biological samples. Comparison of the translation state of glioma cells to non-transformed oligodendrocyte progenitor cells in normal brain identified global alteration of translation in tumor, and specifically of genes involved in cell division and synthetic metabolism. Furthermore, investigation of alteration in steady state translational efficiencies upon loss of PTEN, one of the most frequently mutated and deleted tumor suppressors in glioma, identified differential translation of proteins involved in cellular respiration, canonically regulated by PI3K/Akt signaling, and cellular glycosylation profiles, deregulation of which is known to be associated with tumor progression. Application of the translation efficiency profiling method described here to other biological contexts and conditions would extend our knowledge of the scope and impact of this important mode of gene regulation in complex *in vivo* systems.

## Introduction

The protein state of a cell is subject to multiple layers of regulation. Chromatin accessibility and transcription regulate mRNA concentration, along with factors affecting mRNA stability. Translational regulation controls both global and mRNA specific rates of protein synthesis, and post-translational mechanisms modulate protein half-life and activity. Integration of these multiple modes of regulation controls cell phenotype and dynamic regulation of these factors dictates how a cell responds to stress. Experimental techniques have been developed for measuring the mRNA content of cells, however, methods are less established for studying these other important modes of regulating gene output. This is especially true for the study of complex *in vivo* systems, where cell-type specific resolution is required to identify discrete biology of interacting cells in heterogeneous tissue.

Translation of mRNA into protein is regulated both globally and in a transcript-specific manner, and this process is frequently dysregulated in disease, especially in cancer [1], [2], [3], [4], [5]. Protein translation occurs in three discrete steps: initiation, elongation and termination. Translation initiation is the rate-limiting step and is where most regulation occurs [6]. During canonical cap-dependent translation initiation, the small ribosomal subunit binds to the 7-methyl-GTP cap structure at the 5′ end of an mRNA transcript and scans the 5′ untranslated region (UTR) for the AUG start codon, at which point the large ribosomal subunit is recruited and translation elongation begins [6]. The overall efficiency of this process is regulated by the activity of eukaryotic initiation factors (eIFs), and specific mRNA translation rates are modulated by transcript specific features, such as 5′UTR secondary structure, decoy AUG codons and internal ribosome entry sites (IRESs) [1]. The availability of individual mRNA transcripts to the translation machinery can also be regulated in a sequence-specific manner by microRNAs and RNA-binding proteins [7].

Translational regulation has traditionally been inferred from changes in ribosome-associated mRNAs isolated by sucrose-gradient profiling methods. This technology, however, has limited utility for studying translational regulation *in vivo* because of the cellular complexity of living tissue and the fragility of the polyribosome structure [8], [9]. Here we adapt a method described by Heiman et al. of immunoprecipitating ribosome-bound RNA from genetically-determined cell populations *in vivo* to measure translational regulation in glioma [9]. We demonstrate a broad range of relative ribosome occupancies amongst mRNA species in tumor *in vivo.* Comparison of the translation state of glioma tumor cells to their non-malignant counterpart cells in normal brain revealed widespread alteration of ribosome recruitment efficiencies amongst genes that are associated with malignant behavior. Furthermore, hyperactivity of phosphatidylinositol 3-kinase (PI3K) signaling due to phosphatase and tensin homolog (PTEN) deletion specifically altered ribosome association of mRNAs encoding proteins required for cellular respiration, biology classically associated with PI3K signaling but not previously described to be regulated at the translational level [10], [11], [12]. The tight regulation of ribosome recruitment and the role of translational regulation in affecting discrete classes of genes in glioma demonstrate the importance of this mode of gene regulation. Applied to other disease models and normal tissue contexts, this method will allow for the first time global measurement of translational regulation *in vivo.*

## Results

### *In vivo* Translation Efficiency Measurement in Glioma

**Figure 1:**
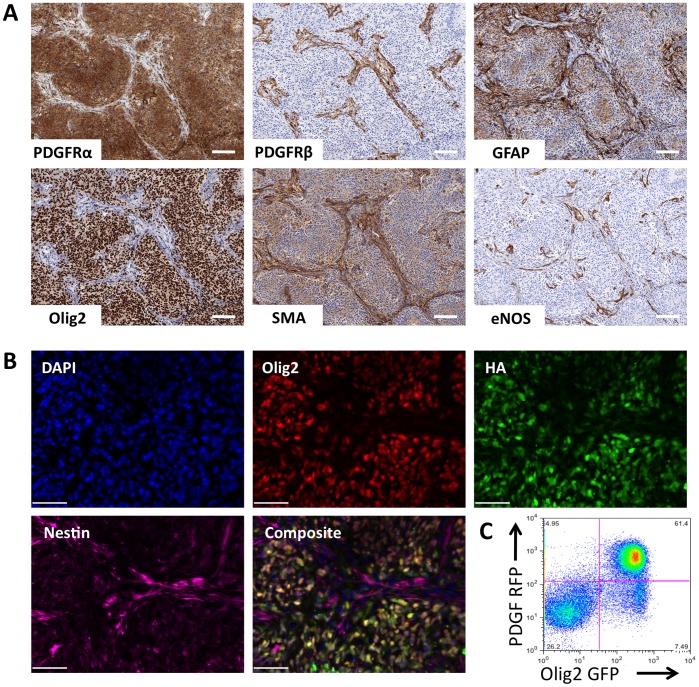
GBM is a heterogeneous tumor composed of multiple cell types, including both tumor and stromal cells. (A) Immunohistochemical staining of mouse PDGF-driven glioma identified multiple tumor regions composed of different cell types. (B) PDGF-HA oncogene expressed from tumor cells colocalized with Olig2 staining by IHC. (C) FACS analysis of tumors generated in Olig2-eGFPL10a mice with PDGF-RFP bicistronic retrovirus identified tumor cells as Olig2+. Scale bar, 100 µm.

To study translational regulation in glioma *in vivo*, we generated high-grade tumors in mice using RCAS tv-a somatic gene transfer technology. [13], [14], [15], [16], [17], [18]. An RCAS retroviral vector expressing the oncogene platelet derived growth factor B (RCAS-PDGF-B) was used to infect cells expressing the RCAS receptor tv-a driven by a Nestin promoter (Ntv-a) in the brains of newborn mice. Experimental mice also harbored somatic deletion of the Ink4a/Arf locus and floxed PTEN alleles (Ntv-a/Ink4a/Arf^−/−/^PTEN^fl/fl^). This GEM of glioma possesses genomic alterations typical of the Proneural subtype of human glioblastoma multiforme (GBM) and closely resembles the human Proneural tumor in gene expression and histology [17], [19]. Like human GBM, PDGFB-driven mouse gliomas were cellularly heterogeneous, composed of transformed cells residing in a complex stromal microenvironment that contributes importantly to tumor progression, but also confounds gene expression study of individual cell populations *in vivo* [20]. Immunohistochemical staining of PDGF-driven gliomas identified eNOS-positive blood vessels surrounded by perivascular regions staining positive for smooth muscle actin (SMA), nestin, and PDGF receptor beta (PDGFRβ), and tumor bulk (TB) regions that are nucleus-dense and stain positive for Olig2 and PDGF receptor alpha (PDGFRα) (Fig. 1A). Astrocytes (GFAP-positive) are numerous and located throughout the tumor (Fig. 1A). Immunofluorescent staining for the hemaglutinin (HA) epitope tag on the PDGF protein used to generate gliomas revealed that most, if not all, PDGF-HA expressing tumor cells colocalized with Olig2 expression in the TB regions (Fig. 1B). FACS analysis of gliomas generated with a bicistronic RCAS vector expressing both PDGF and RFP in mice expressing GFP driven by the Olig2 promoter confirmed the identity of oncogene-expressing glioma cells as Olig2-positive (Fig. 1C).

The ability to distinguish tumor cells from tumor stroma by Olig2 expression allowed us to develop a method for global quantification of translation efficiency for individual mRNAs in glioma cells residing in their native tumor microenvironment. PDGF-induced gliomas were generated in mice transgenic for the gene fusion of the large ribosome subunit protein L10a with eGFP under the transcriptional control of an Olig2 BAC promoter [21], [22]. In these tumors, eGFP-L10a expression was restricted to Olig2+cells in the tumor (Fig. S1A). This cell-type specific expression of eGFP-L10a enabled immunoprecipitation of ribosome-bound translating mRNA with anti-GFP-conjugated beads from only the Olig2+tumor cells by a method previously described [9], [21]. GFP expression also allowed flow cytometric sorting of Olig2+tumor cells to isolate total cellular RNA from the same cell population. The translation efficiency (TE) for each mRNA transcript could then be expressed as a ratio of the expression values from translating ribosome-bound RNA and total cellular RNA (IP/Total). Since translation initiation has been shown to be the rate-limiting step of translation, the TE ratio should be a good proxy for the relative efficiency of protein production from individual transcripts. The TE measurement for each biological sample was then regarded as a discrete biological value because both pools of RNA were obtained as pairs from the same tumor.

**Figure 2:**
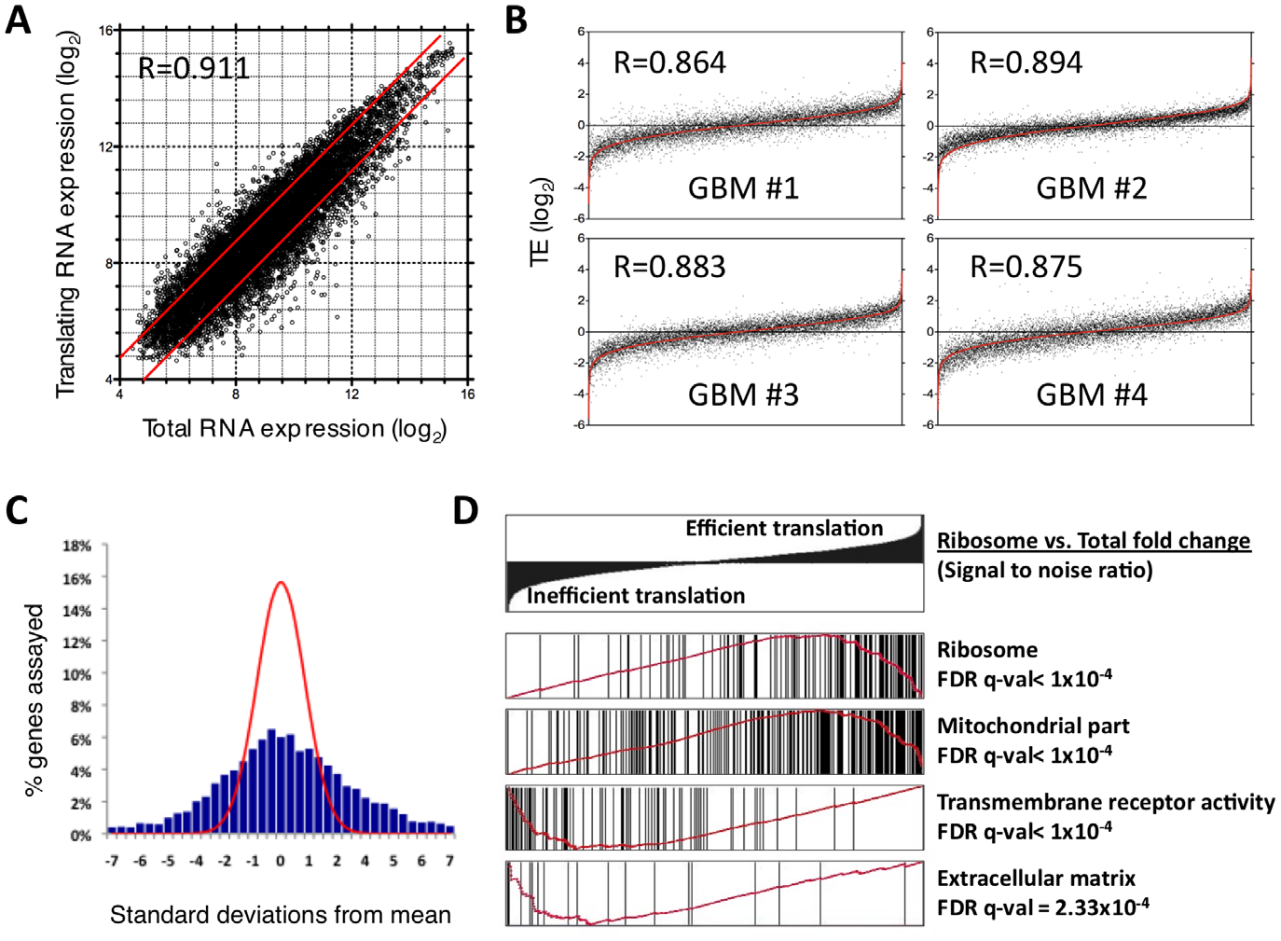
*In vivo* quantification of ribosome-bound and total RNA levels revealed a broad range of ribosome recruitment efficiencies amongst mRNA transcripts. (A) Distribution of mRNA expression in ribosome-bound and total RNA pools from PDGF-driven glioma identified differential TE (N = 4). (B) TE values for each biological replicate (black points) plotted with the average of the other three replicates (red line) demonstrated reproducibility of measurements. (C) Signal-to-noise ratios of TE measurements (blue bars) identified range of high confidence measurements relative to a normal distribution (red line). (D) GSEA identified statistical overrepresentation of defined gene ontologies amongst efficiently and inefficiently translated genes. Black bars represent distribution of mRNAs from indicated geneset amongst all genes ranked by signal to noise ratio (top panel). Red line represents GSEA output enrichment score. R = Pearson correlation coefficient.

Ribosome-bound and total cellular RNA samples from Olig2+mouse glioma cells were quantified by expression microarray (Fig. 2A). The specificity of the TE measurement could be demonstrated with the mRNA transcript Xist, which is highly transcribed from the silenced X-chromosome in females and mediates X-inactivation by coating the silenced chromosome without being translated [23]. Xist mRNA transcript was abundant in the total RNA pool in tumors generated in female mice, but entirely absent from ribosome-bound RNA (Fig. S1B). Xist transcript was not present in either pool of RNA from tumors generated in male mice (Fig. S1B).

The translation efficiency for individual transcripts was highly reproducible across four independent tumor samples. Pearson correlation coefficients of relative TE for each biological replicate compared to the average of the other three tumor samples exceeded 0.86 for all four comparisons (Fig. 2B). Furthermore, distribution of signal to noise of TE values greatly exceeded normal error, with 1,177 probes (12.2%) measuring signal to noise ratios greater than five (Fig. 2C). Reliability of TE measurements across the range of expression could be demonstrated by comparison of TE values for the 2,149 genes with multiple unique probes. Multiple probes to the same gene were found to measure very similar TE across the range of microarray probe values (Fig. S2).

The TEs of mRNA transcripts in glioma tumor cells *in vivo* demonstrated more than a 600-fold range of translation efficiency amongst the 9,653 transcript probes with detectable expression by microarray, illustrating the substantial contribution of translational regulation to gene output. More than 60% (5,963) of all genes were identified as differentially represented in the total RNA and translating RNA pools using a conservative multiple testing correction filter of FDR<0.05 (Dataset S1). Of the probes measuring significant translational regulation, 1065 were at least 2-fold more prevalent in the translating pool (efficiently translated) and 1156 probes more than 2-fold less efficiently translated than average (Fig. 2A; Dataset S1).

The high degree of similarity of TE measures between individual tumors suggests tight transcript-specific control of translation to modify gene output and regulate cell behavior. Gene set enrichment analysis (GSEA) identified overrepresentation of gene ontology (GO) categories amongst genes translated at different rates, implying that differential TE regulates specific cell behaviors [24], [25]. RNAs encoding ribosomal and mitochondrial proteins were amongst the most efficiently translated messages in PDGF-driven mouse glioma (Fig. 2D, Dataset S1), concordant with translation efficiency measures determined by ribosome footprint profiling for yeast cells collected during log-phase growth [26]. GO categories encompassing transmembrane receptor activity and extracellular matrix components were amongst the least efficiently translated mRNAs (Fig. 2D).

While the data above describes the static translation state in PDGF-driven glioma, this method enables comparison of the translation state of genetically-defined cell types to characterize cell-type specific differences in translational regulation. We therefore sought to compare the translation state of the transformed glioma cells with their untransformed normal cell counterparts.

### Dysregulation of Translation in Neoplastic Transformation

Dysregulation of protein synthesis is a common feature of cancer that modulates gene output and may contribute to malignant phenotype [2], [4]. We had previously identified alterations of translation in cultured glial cells upon transformation with activated Ras and Akt [5]. However, any *in vitro* culture system will naturally fail to recapitulate the complex set of extracellular signals and cues comprising the *in vivo* microenvironment. Hierarchical clustering of PDGF-driven mouse glioma gene expression profiles with various mouse neural cell types characterized these tumor cells as most similar to oligodendrocyte progenitor cells (OPCs) in the normal brain that also express Olig2 [27]. This finding is consistent with the oligodendroglial phenotype of the Proneural subset of human gliomas that the PDGF-driven mouse model most closely resembles [17]. Therefore, we were able to apply the *in vivo* TE profiling strategy to compare the translation state of transformed glioma cells *in vivo* with that of their non-transformed OPC counterparts, offering insight into the dysregulation of translation that occurs in glioma *in vivo*.

**Figure 3:**
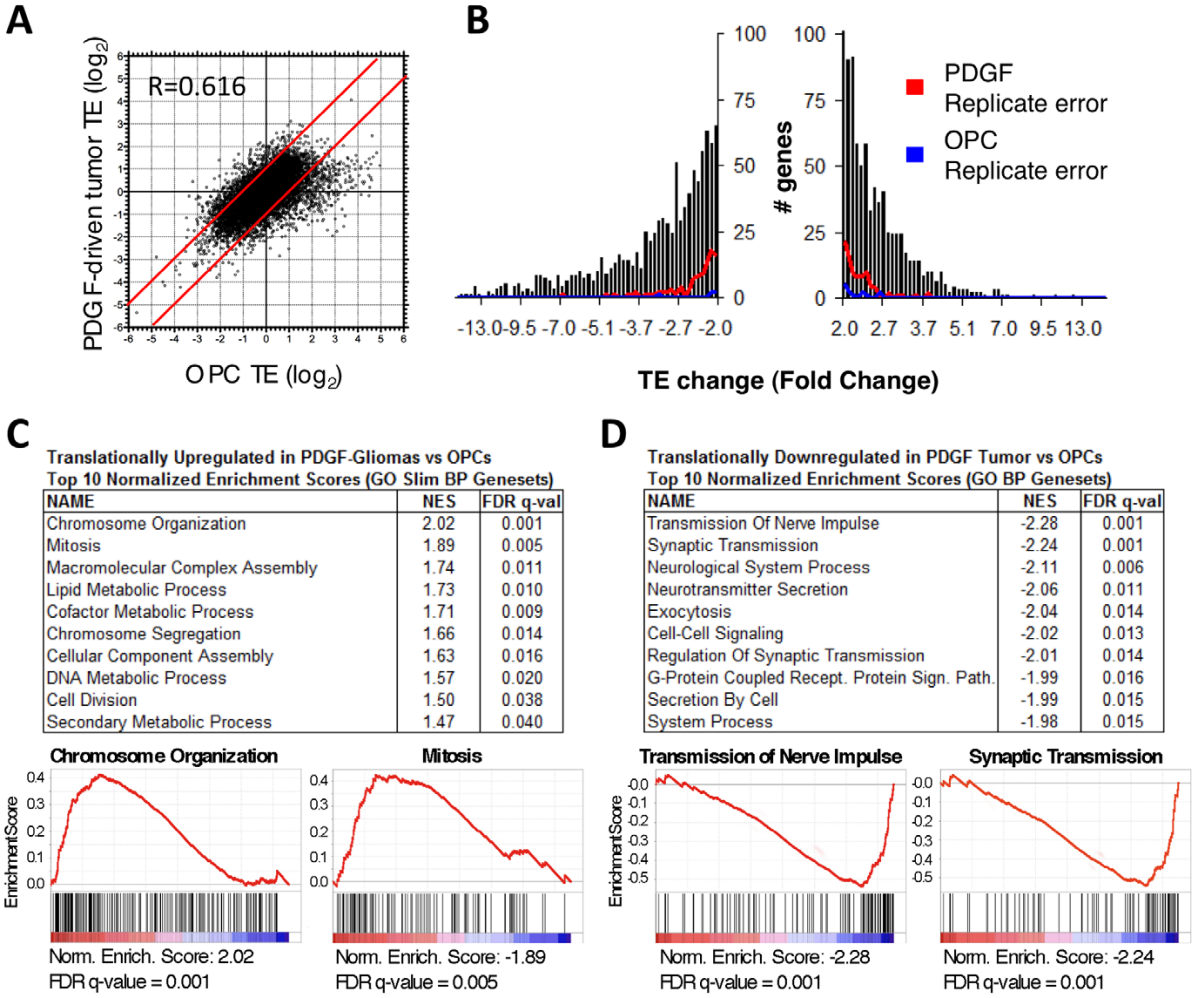
Translation efficiency was altered in glioma compared to normal brain OPCs. (A) Changes in ribosome-bound mRNA correlated somewhat with changes in total cellular RNA between tumor and normal brain OPCs (N = 4). (B) Alterations in TE between tumor and normal brain were reproducible, exceeding average replicate error. (C) Genesets associated with cell division and biosynthetic pathways predominated amongst GOSlim Biological Process genesets most enriched for transcripts translationally upregulated in Olig2+tumor cells compared to normal brain OPCs. Enrichment plots for chromosome organization and mitosis genesets shown. (D) Genesets associated with synaptic signaling predominated amongst GO Biological Process genesets most enriched for transcripts translationally downregulated in Olig2+tumor cells compared to normal brain OPCs. Enrichment plots for transmission of nerve impulse and synaptic transmission shown. NES = Normalized Enrichment Score. R = Pearson correlation coefficient.

As in tumor, relative TE values in Olig2-positive OPCs in adult normal brain varied widely amongst mRNA species and were highly correlated across replicates (average R = 0.959) (Fig. S3A, S3B; Dataset S2). Comparison of relative ribosome occupancies of tumor and normal Olig2-positive cells revealed a positive correlation of 0.616, demonstrating substantial similarity in the measured translation states of the two cell populations (Fig. 3A). Many genes, however, were translated with altered efficiency in tumor compared to normal brain OPCs (3,902 probes FDR<0.05; 40.4%) (Dataset S2), with 799 probes detecting greater than 2-fold increases in TE in tumor, and 912 probes detecting more than 2-fold decreases in TE (Fig. 3B; Fig. S3D; Dataset S2).

Discrete biological functions were enriched amongst the translationally altered transcripts. GSEA identified significantly translationally upregulated GO genesets that fell into two broad categories: cell division and biosynthetic pathways. Genesets for chromosome organization and mitosis were the most upregulated genesets, and chromosome segregation and cell division were also among the genesets meeting a 0.05 FDR cutoff (Fig. 3C). Accordingly, genes involved in chromatin packing and cytokinesis were among the most significantly translationally upregulated transcripts, with histones and tubulins together comprising 9 of the top 35 most upregulated transcripts (Hist1 h3a 10.9-fold; Hist1 h3e 5.9-fold; Hist1 h3d 5.7-fold; Hist1 h4 m 5.6-fold; Hist1 h4d 5.3-fold; Tuba3b 5.2-fold; Tubb4 4.9-fold; Tuba3a 4.9-fold; Hist1 h4f 4.6-fold). Numerous major cell cycle regulators were also significantly translationally upregulated (CDCA7 9.0-fold; cyclin D2 4.1-fold; cyclin B1 2.7-fold; CDK2 2.0-fold), as well as multiple minichromosome maintenance proteins required for licensing DNA for synthesis (MCM6 3.0-fold; MCM10 2.8-fold; MCM2 2.7-fold). In addition, most major biosynthetic processes were translationally upregulated, including lipid metabolism, cofactor metabolism, DNA metabolism, and secondary metabolic processes (Fig. 3C). The upregulation of these synthetic pathways provides structural components needed for rapid cellular replication.

The genesets that were significantly downregulated translationally were associated with neural signaling, and specifically processes involved in pre-synaptic signaling (Fig. 3D, Fig. S3D). Some OPCs have been shown to give rise to neurons, and the translational downregulation of synaptic transmission related genes may be reflective of a lack of neuronal differentiation in the PDGF-driven tumor cell population [28], [29]. Lack of differentiation is a general feature of cancer and is indicative of higher grade and worse prognosis in gliomas [30], [31]. The role of translational regulation in contributing to proliferative capacity and cell fate determination is of critical scientific and therapeutic interest and warrants further investigation into the mechanisms of this regulation.

### PTEN Loss Reduces the Ribosome Occupancy of Transcripts Associated with Cellular Respiration in Glioma

The PI3K pathway is an important transduction pathway downstream of receptor tyrosine kinase signaling that is frequently dysregulated in cancer [32]. PI3K catalyzes the conversion of phosphatidylinositol (4,5) bisphosphate (PIP_2_) to phosphatidylinositol (3,4,5) bisphosphate (PIP_3_), which activates Akt and downstream mTORC1 signaling, a well-known regulator of protein translation [33]. PTEN opposes PI3K function and is frequently altered in GBM, with 36% of tumors exhibiting homozygous deletion or mutation [34]. In the Proneural subgroup of human GBMs, 69% of tumors had homozygous or hemizygous deletion of PTEN, and PTEN mutations were found in 16% of tumors [17]. Deletion of PTEN in mouse PDGF-driven gliomas results in increased stem-like phenotype, reduced tumor latency, and histologically more aggressive tumors featuring increased pseudopallisading necrosis [35], [36]. To determine if loss of PTEN altered the homeostatic translation state in glioma, we delivered RCAS-Cre virus along with RCAS-PDGFB into Ntv-a/Ink4a/Arf^−/−/^PTEN^fl/fl^ mice and collected ribosome-bound and total cellular RNA from Olig2+cells. The resulting tumors exhibited an overall expression pattern similar to that of PTEN WT tumors (Fig. S5), but had undetectable levels of PTEN in the tumor bulk regions and elevated levels of phosphorylated Akt, a marker of PI3K activity, and elevated phosphorylated ribosomal protein S6, a marker of mTORC1 pathway activation (Fig. S4A and S4B).

**Figure 4:**
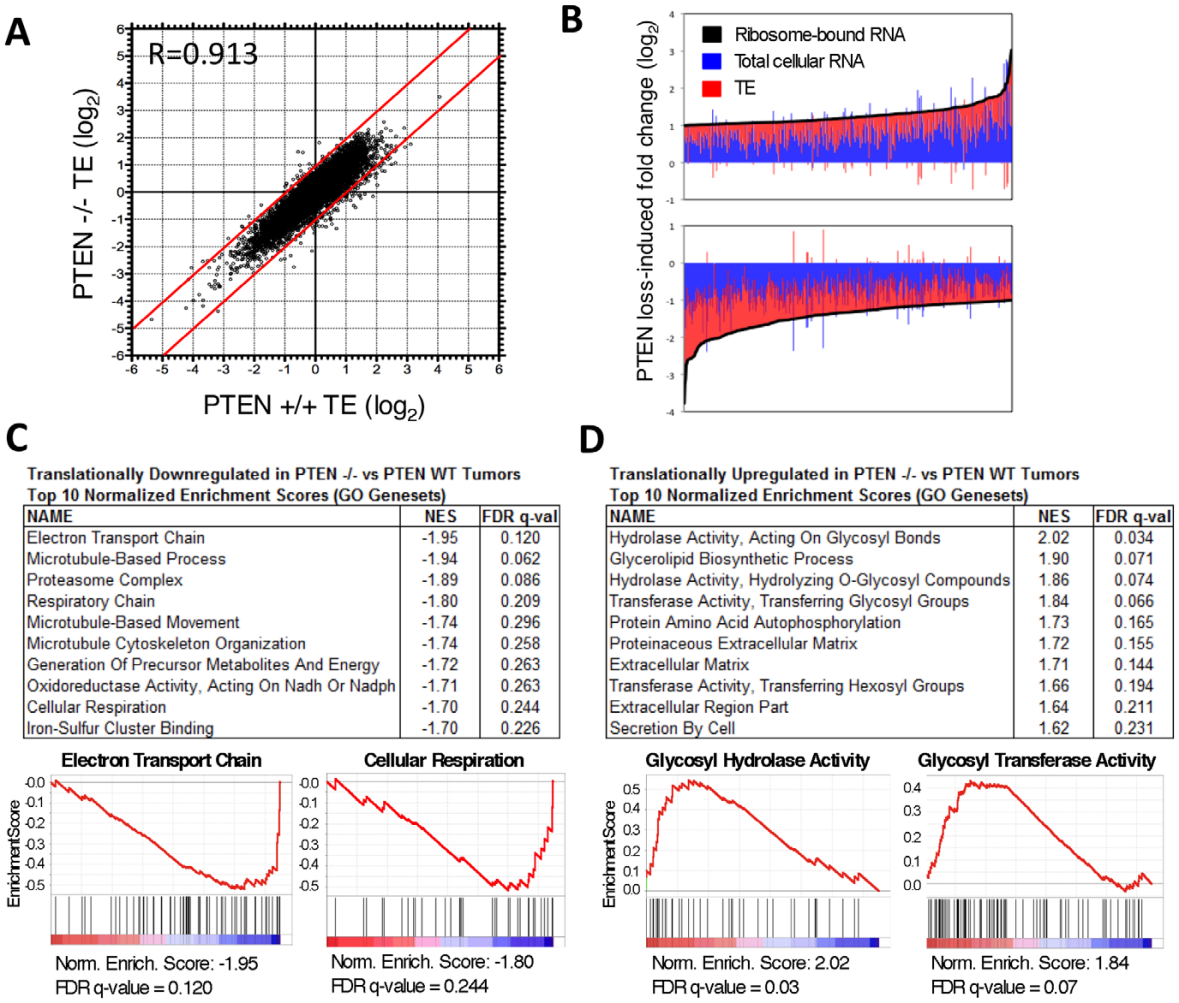
PTEN loss altered the translation state of glioma cells. (A) TE measurements of PTEN WT and PTEN deleted tumors were very similar (N = 4). (B) Changes in both total RNA and TE contributed to alteration in ribosome-bound RNA caused by PTEN loss. (C) GO genesets associated with oxidative phosphorylation were amongst those most enriched for transcripts translationally downregulated in PTEN-deleted vs PTEN WT tumors. Enrichment plots for electron transport chain and cellular respiration genesets shown. (D) Genesets associated with glycolysis predominated amongst GO genesets most enriched for transcripts translationally upregulated in PTEN-deleted vs PTEN WT tumors. Enrichment plots shown for genesets for hydrolase activity acting on glycosyl bonds and transferase activity transferring glycosyl bonds. NES = Normalized Enrichment Score. R = Pearson correlation coefficient.

Analysis of PTEN^−/−^ gliomas relative to PTEN^fl/fl^ gliomas demonstrated a high degree of similarity in TE between the two tumor types, in contrast to the widespread changes observed between tumor and normal OPCs (Fig. 4A). Reproducible changes in the transcriptome and translatome, however, were detected upon PTEN loss in glioma. 914 genes were more than 2-fold differentially expressed in the IP fraction and met an FDR cutoff of 0.05, resulting from alteration of both total mRNA content and translational regulation (Fig. 4B; Fig. S4E; Dataset S3). Of these most changed genes (>2-fold in ribosome-bound mRNA), alteration of TE accounted for more than 40% of gene expression changes (Fig. 4B). Somewhat surprisingly, differential translation contributed most significantly to downregulated gene expression, with 54 probes measuring greater than 2-fold reduction in TE, and only 4 genes exhibiting greater than 2-fold increased TE upon PTEN loss (FDR<0.05) (Table 1; Table 2; Fig. S4E). These differences we observe in gene expression likely results directly from the increased PI3K/Akt/mTORC1 activity in the PTEN-deleted tumors in addition to differences in tumor initiation and evolution upon PTEN deletion.

**Table 1:** PDGF+Cre vs. PDGF only tumors; genes with >2-fold downregulated TE and FDR<0.05.

		PDGF+Cre vs. PDGFLog2 Change		
Gene Symbol	Description	PC-P IP	PC-P Total	PC-P TE
**UBE3A**	ubiquitin protein ligase E3A	−2.71	−0.66	−2.05
**CCT5**	chaperonin containing Tcp1, subunit 5 (epsilon)	−3.26	−1.22	−2.03
**SLC3A2**	solute carrier family 3 (activators of dibasic and neutral amino acid transport), member 2	−2.04	−0.02	−2.02
**EID1**	EP300 interacting inhibitor of differentiation 1	−3.77	−2.00	−1.77
**PCNA**	proliferating cell nuclear antigen	−2.61	−0.86	−1.75
**IDI1**	isopentenyl-diphosphate delta isomerase	−1.91	−0.20	−1.71
**RSPRY1**	ring finger and SPRY domain containing 1	−2.37	−0.66	−1.71
**LOC100048530**	similar to coiled-coil domain containing 72	−2.25	−0.72	−1.53
**FABP7**	fatty acid binding protein 7, brain	−2.60	−1.10	−1.50
**SOX2**	SRY-box containing gene 2	−2.73	−1.27	−1.46
**LOC668837**	similar to ATP synthase, H+ transporting, mitochondrial F0 complex	−2.63	−1.16	−1.46
**TAF9**	TAF9 RNA polymerase II, TATA box binding protein (TBP)-associated factor	−1.86	−0.43	−1.43
**ABCE1**	ATP-binding cassette, sub-family E (OABP), member 1	−1.65	−0.24	−1.40
**HUWE1**	HECT, UBA and WWE domain containing 1	−2.21	−0.82	−1.40
**GFRA1**	glial cell line derived neurotrophic factor family receptor alpha 1	−0.94	0.43	−1.37
**NDUFB4**	NADH dehydrogenase (ubiquinone) 1 beta subcomplex 4	−2.03	−0.68	−1.35
**PIK3CA**	phosphatidylinositol 3-kinase, catalytic, alpha polypeptide	−1.77	−0.42	−1.34
**UBE2N**	ubiquitin-conjugating enzyme E2N	−2.04	−0.72	−1.33
**STMN1**	stathmin 1	−2.21	−0.89	−1.32
**UBTD2**	ubiquitin domain containing 2	−1.89	−0.61	−1.29
**KLHL13**	kelch-like 13	−1.65	−0.37	−1.28
**ZFP647**	zinc finger protein 647	−1.74	−0.46	−1.28
**USMG5**	upregulated during skeletal muscle growth 5	−2.03	−0.76	−1.28
**2610528E23RIK**	RIKEN cDNA 2610528E23 gene	−1.73	−0.45	−1.27
**JAM3**	junction adhesion molecule 3	−2.31	−1.06	−1.25
**MXI1**	Max interacting protein 1	−2.59	−1.34	−1.24
**HNRNPA2B1**	heterogeneous nuclear ribonucleoprotein A2/B1	−2.51	−1.30	−1.22
**SNX1**	sorting nexin 1	−2.12	−0.94	−1.19
**SPCS2**	signal peptidase complex subunit 2 homolog	−1.78	−0.60	−1.18
**NDRG2**	N-myc downstream regulated gene 2	−1.39	−0.21	−1.18
**SNX3**	sorting nexin 3	−2.04	−0.86	−1.17
**OTTMUSG00000007855**	predicted gene, OTTMUSG00000007855	−2.58	−1.41	−1.17
**SDHD**	succinate dehydrogenase complex, subunit D	−1.44	−0.28	−1.16
**1500003O22RIK**	RIKEN cDNA 1500003O22 gene	−1.61	−0.47	−1.14
**TUG1**	taurine upregulated gene 1	−2.05	−0.93	−1.13
**PCMT1**	protein-L-isoaspartate (D-aspartate) O-methyltransferase 1	−1.82	−0.69	−1.12
**CHMP5**	chromatin modifying protein 5	−2.08	−0.96	−1.12
**SLAIN1**	SLAIN motif family, member 1	−0.78	0.34	−1.12
**CYCS**	cytochrome c	−2.06	−0.94	−1.12
**RP23-297J14.5**	similar to oxidative stress responsive 1	−1.51	−0.40	−1.11
**ZKSCAN3**	zinc finger with KRAB and SCAN domains 3	−2.02	−0.92	−1.11
**LOC100041703**	similar to G protein gamma-5 subunit	−2.02	−0.93	−1.09
**CRYZL1**	crystallin, zeta (quinone reductase)-like 1	−1.68	−0.60	−1.08
**RSU1**	Ras suppressor protein 1	−2.06	−0.98	−1.08
**LOC100046796**	similar to polymerase (RNA) II (DNA directed) polypeptide K	−1.35	−0.28	−1.07
**ST13**	suppression of tumorigenicity 13	−1.27	−0.22	−1.05
**LOC100044087**	similar to brain protein 44	−2.16	−1.10	−1.05
**GTF2E2**	general transcription factor II E, polypeptide 2 (beta subunit)	−1.66	−0.61	−1.05
**SPRR2A**	small proline-rich protein 2A	−1.46	−0.41	−1.05
**ZFP161**	zinc finger protein 161	−1.85	−0.81	−1.04
**PDCD5**	programmed cell death 5	−1.74	−0.72	−1.02
**CFL2**	cofilin 2, muscle	−1.87	−0.85	−1.02
**NIT2**	nitrilase family, member 2	−1.76	−0.74	−1.02
**COX7B**	cytochrome c oxidase subunit VIIb	−1.60	−0.59	−1.01

**Table 2:** PDGF+Cre vs. PDGF only tumors; genes with >2-fold upregulated TE and FDR<0.05.

		PDGF+Cre vs. PDGFLog2 Change		
Gene Symbol	Description	PC-P IP	PC-P Total	PC-P RRO
**GALE**	galactose-4-epimerase, UDP	1.38	0.28	1.10
**D9ERTD402E**	DNA segment, Chr 9, ERATO Doi 402, expressed	1.59	0.40	1.19
**TTC14**	tetratricopeptide repeat domain 14	0.66	−0.64	1.30
**KLHDC9**	kelch domain containing 9	1.51	−0.19	1.70

The genes translationally downregulated in PTEN null cells were significantly enriched for biological functions canonically associated with PI3K/Akt pathway activity. The catalytic subunit of PI3K, PIK3CA, was 2.5-fold downregulated translationally in PTEN null tumors. Deletion of PIK3CA results in reduced PI3K activity and Akt signaling, therefore it is possible that translational downregulation of PIK3CA represents a feedback mechanism to attenuate PI3K signaling in the absence of PTEN phosphatase [37].

Activation of the PI3K/Akt/mTOR signaling axis is well known to induce a shift in cellular metabolism from oxidative phosphorylation to glycolysis (Warburg effect) [12], [38]. The GO geneset describing the electron transport chain was the most enriched geneset amongst translationally downregulated transcripts upon PTEN deletion (Fig. 4C). GO genesets for the respiratory chain, generation of precursor metabolites and energy, oxidoreductase activity acting on NADP or NADPH, and cellular respiration were similarly enriched. Amongst the most translationally downregulated individual transcripts in PTEN deleted tumors were critical components of the mitochondrial electron transport chain, including Complex I NADH dehydrogenase (NDUFB4), Complex II succinate dehydrogenase (SDHD) and Complex IV cytochrome c oxidase (COX7b) and cytochrome c (CYCS) (Table 1). Translational reduction of these important electron transport chain enzymes would be expected to reduce the cellular capacity for oxidative phosphorylation, consistent with the overall metabolic shift towards glycolysis observed in PTEN null cells. Since hypoxia is associated with a shift to glycolysis, the increased pseudopallisading nature of the PTEN deleted tumors may be directly or indirectly associated with the translational alterations seen here.

Despite the smaller fold changes in TE amongst the most translationally upregulated transcripts in PTEN deleted tumors, GSEA identified genesets enriched with similar significance amongst translationally upregulated transcripts. The GO genesets most enriched by PTEN deletion were associated with hydrolysis and transfer of glycosyl groups (Fig. 4D). Secreted and extracellular proteins and lipids are the main substrates for glycosylation. Accordingly, translationally upregulated transcripts were also enriched for extracellular matrix and secretion genesets, as well as hexosyl modifications. Taken together, these data suggest that changes in translation efficiency contribute to an altered extracellular glycosylation profile in PTEN-deleted tumors. Proteoglycans comprise much of the extracellular matrix, and many of the proteins involved in sensing the extracellular environment, including growth factor receptors, integrins, and cadherins, are subject to glycosylation. Glycosylation can affect binding properties, half-life, and localization of these biomolecules. As such, alterations in glycosylation have widespread effects on cell behavior; there is evidence that alterations in glycosylation affect tumor growth, migration, invasion, and angiogenesis [39], [40]. Indeed, aberrant glycosylation is known to be correlated with tumor aggressiveness, progression, and survival rates in various cancers [41], [42]. Alterations of glycosylation via translational upregulation may be an underappreciated mechanism by which PTEN modulates tumor aggressiveness.

## Discussion

Regulation of protein levels that dictate cell phenotype and behavior occurs by multiple mechanisms in the cell. Messenger RNA content is most commonly used to approximate gene expression because of the availability of genome-wide microarray and sequencing technologies. This approach, however, ignores important post-transcriptional regulatory mechanisms that have profound impact on gene expression. The technical challenges of interrogating post-transcriptional gene regulation are compounded in complex *in vivo* settings in which cells are often few and tissue is heterogeneous. Here we describe a sensitive method for measuring translational regulation in genetically-defined cell populations *in vivo.* We identified substantial differences in the abundance of mRNA transcripts in total cellular RNA and those bound to ribosomes in both glioma tumor cells and normal brain OPCs, demonstrating the important contribution of translational regulation to gene expression. Furthermore, differentially translated messages were functionally coherent, with transcripts encoding mitochondrial and ribosomal proteins enriched amongst the most efficiently translated mRNAs.

Comparison of TE measurements in glioma cells to their non-tumor cell equivalent OPCs in normal brain uniquely allowed insight into the dysregulation of translation that occurs in tumor cells in a relevant *in vivo* context. We identified broad alterations in TE in glioma that contribute significantly to gene expression changes. Many of the translationally upregulated mRNAs encode proteins that promote the proliferative capacity of the tumor cells by enhancing translation of proteins critical for DNA synthesis, chromosome packaging and segregation and lipid metabolism. The translationally downregulated mRNAs are enriched for processes of synaptic signaling, reflecting the lack of differentiation of the tumor cells. We further identified alteration of translation of very specific transcripts upon PTEN deletion that are required for oxidative phosphorylation, a biological function classically associated with Akt pathway activity and a hallmark of cancer. We also identified enhanced translation of transcripts associated with glycosylation modification, whose dysregulation is associated with more aggressive cancers. The mechanisms of translational dysregulation in cancer and as a result of tumor suppressor loss are important areas of future investigation that may identify new therapeutic targets. Pharmacological modulation of transcriptional mechanisms have remained elusive, however, modulation of protein translation may be more amenable to therapeutic intervention and offer new approaches to targeting dysregulated gene expression in cancer.

In the present study, we characterized the homeostatic translation state of glioma cells and OPCs *in vivo*. Investigation of the variation in ribosome occupancy of specific transcripts between different cell types and at different stages of development would offer a broader view of the role of translational regulation in contributing to cellular diversity. This method can be further applied to study dynamic regulation of translation in response to stress or therapy. Translational changes may be more efficient than transcriptional regulation for rapidly altering the cellular concentration of proteins, and dynamic changes in translational efficiency have been identified in response to a variety of cellular stresses [1], [43]. In fact, translational changes have been reported to be much more significant than transcriptional changes in glioma cell lines exposed to ionizing radiation, the first-line therapy for patients with glioma [44]. A better understanding of the translational response to cellular stresses would enhance our understanding of dynamic gene expression regulation and may have important clinical implications. The TE profiling method presented here provides a powerful tool for interrogating translational regulation in complex *in vivo* systems and may lead to a more complete understanding of homeostatic and dynamic gene expression control in different cell types and in changing conditions.

## Materials and Methods

### Mice and Generation of Murine Gliomas

Ntv-a/Ink4a/Arf^−/−/^PTEN^fl/fl^ mice, described previously [45], were bred with Olig2 bacTRAP mice [21] (gift from Dr. Nathaniel Heintz) and gliomas were generated by RCAS-mediated retroviral transduction by a method previously described [13], [46]. Injected mice were aged until they became symptomatic (lethargy, weight loss, macrocephaly). Normal brain OPCs were collected from non-injected 4–8 week old Ntv-a/Ink4a/Arf^−/−/^PTEN^fl/fl^/Olig2-eGFP-L10a mice. All animal studies were done in accordance with protocols approved by the Institutional Animal Care and Use Committee of Memorial Sloan-Kettering Cancer Center and followed National Institutes of Health guidelines for animal welfare. Genotyping primers will be provided on request.

### Histology, Immunohistochemistry and Immunofluorescence

Tissues were fixed in 10% neutral buffered formalin and imbedded in paraffin according to standard procedures [47]. Immunohistochemical staining of tissue sections was performed on the Discovery XT automated staining system (Ventana Medical Systems). The following antibodies were diluted in PBS/2%BSA: PDGFRβ 1∶300 (Cell Signaling 3169), PDGFRα 1∶300 (Cell Signaling 3174), PTEN 1∶100 (Cell Signaling 9559), pS6 S235/236 1:300 (Cell Signaling 2211), Olig2 1:300 (Millipore AB9610), SMA 1∶100 (Dako M0851), GFAP 1∶5000 (Dako M0761) and eNOS 1∶100 (BD Biosciences 610296). Immunoflourescent staining was performed according to methods previously described [48] using the following antibodies: Olig2 1:250 (Millipore AB9610), Nestin 1∶100 (BD Biosciences 556309) and HA 1∶100 (Roche 11867423001).

### Collection and Labeling of Ribosome-bound and Total Cellular RNA

For collection of total cellular RNA, normal brain or grossly-dissected tumor tissue was dissociated into a single cell suspension with papain by a method previously described [35] and GFP+cells were collected by FACS (Becton-Dickinson Aria Cell Sorter). Sorted cells were collected with Trizol-LS (Invitrogen), chloroform extracted, precipitated with sodium acetate in isopropanol overnight, and purified with Qiagen RNeasy MinElute Cleanup Kit. Ribosome-bound RNA was immunoprecipitated with anti-GFP conjugated magnetic beads from mechanically-homogenized tissue as previously described [9] and purified as above. Tissue samples were divided equally for total and ribosome-bound RNA to generate paired datasets. 200 ng of RNA was amplified and biotin-labeled according to manufacturer protocol (Ambion AMIL1791) and hybridized to Illumina MouseRef-8 v2.0 Expression BeadChips (Rockefeller University Genomics Resource Center). Each biological replicate represents one tumor sample or 5 pooled normal brains.

### Microarray Analysis and Gene Set Enrichment

Microarrays were analyzed using Illumina GenomeStudio Gene Expression Software. A detection p-value filter of 0.05 was applied to background subtracted expression values and data was exported to Partek Genomics Suite software for quantile normalization and log_2_ transformation. TE values were calculated as IP minus Total log_2_ expression values for each biological replicate. Statistical significance was determined by 1-way ANOVA testing with FDR<0.05 using Partek software. Statistical analysis of standard deviation and Pearson correlation were performed using GraphPad Prism v5.0 software or Microsoft Excel. Gene set enrichment analysis was performed with the javaGSEA Desktop Application using 1000 gene set permutations and all other default settings [24]. Gene ontology genesets were created using GO or GOSlim ontologies and mouse annotation files downloaded on 4/13/11 [25].

## Supporting Information

Figure S1 ***In vivo*** **quantification of ribosome-bound and total RNA levels in PDGF-driven mouse glioma.** (A) Olig2-eGFP-L10a expression faithfully reported endogenous Olig2 expression in tumor. (B) Xist mRNA was abundant in total RNA from tumors generated in female mice, but absent from the ribosome-bound fraction (SD bars shown).
(TIF)

Figure S2 **Multiple probes against the same gene identified similar TE measurements across the full range of expression values in total RNA.** Average percent difference in measures TE between probe and gene average were binned by expression value in total RNA for (A) PDGF-driven mouse glioma, (B) normal brain OPCs and (C) PDGF-driven mouse glioma with PTEN deleted.
(TIF)

Figure S3 **TE measurements in normal brain OPCs. (A) Distribution of mRNA expression in ribosome-bound and total RNA pools from normal brain OPCs identified differential ribosome recruitment efficiencies.** (B) TE values for each biological replicate (black points) plotted with the average of the other three replicates (red line) demonstrated reproducibility of measurements. (C) Signal to noise ratios of TE measurements (blue bars) identified range of high confidence measurements relative to normal distribution (red line). (D) Venn diagram of probes changed greater than 2-fold (and FDR<0.05) between tumor and normal Olig2+cells in ribosome-bound RNA (IP), total cellular RNA (Total) and TE. Pearson correlation coefficients (R) are represented.
(TIF)

Figure S4 **TE measurements in PDGF-driven glioma with PTEN deleted.** (A) Immunohistochemical staining of tumor sections demonstrated the absence of PTEN protein and increased p-S6 positivity in mice receiving Cre virus in addition to PDGF. (B) Western blot of tumor lysates from mouse glioma demonstrates increased phosphorylation of Akt (T308) and S6 (S235/236) in tumors with PTEN deleted compared to tumors expressing PTEN. (C) Distribution of mRNA expression in ribosome-bound and total RNA pools from PDGF+Cre tumors identified differential translational efficiencies. (D) TE values for each biological replicate (black points) plotted with the average of the other three replicates (red line) demonstrated reproducibility of measurements. (E) Signal to noise ratios of TE measurements (blue bars) identified range of high confidence measurements relative to normal distribution (red line). (F) Venn diagram of probes changed greater than 2-fold (and FDR<0.05) between PTEN^fl/fl^ and PTEN^−/−^ Olig2+cells in ribosome-bound RNA (IP), total cellular RNA (Total) and TE. (G) mRNAs involved in cellular respiration and encoding components of the electron transport chain were translationally downregulated upon PTEN loss. Pearson correlation coefficients (R) are represented.
(TIF)

Figure S5 **Hierachical clustering of normalized microarray expression data from (A) total cellular RNA and (B) ribosome-bound RNA extracted from Olig2+cells in normal brain and PDGF-driven glioma demonstrates the high degree of similarity of PTEN-expressing and PTEN-deleted tumors compared to normal brain OPCs.** Hierachical clustering performed on transcripts significantly different by ANOVA at 0.05 FDR.
(TIF)

Dataset S1 **PDGF-driven glioma; IP, Total and TE values.**
(XLS)

Dataset S2 **PDGF-driven glioma compared to normal brain OPCs; IP, Total and TE values.**
(XLS)

Dataset S3 **PTEN^+/+^ PDGF-driven glioma compared to PTEN^−/−^ PDGF-driven glioma; IP, Total and TE values.**
(XLS)
