# Supplementary figures and images for: Identification of Global Alteration of Translational Regulation in Glioma In Vivo
Source: PLoS One. 2012 Oct 3;7(10):e46965. doi: 10.1371/journal.pone.0046965 (PMC3463531; doi:10.1371/journal.pone.0046965)

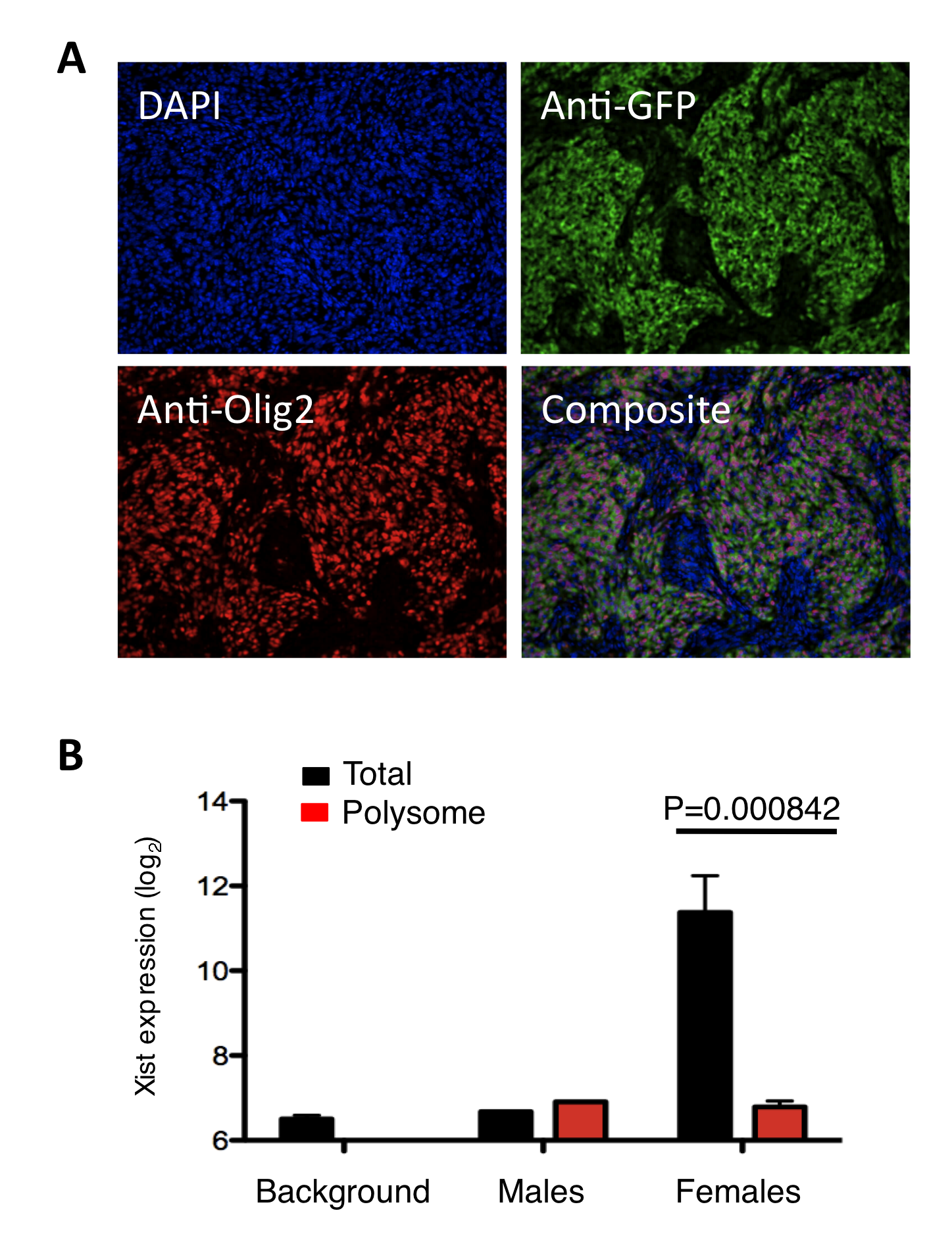

Supplement: Figure S1 — In vivo quantification of ribosome-bound and total RNA levels in PDGF-driven mouse glioma. (A) Olig2-eGFP-L10a expression faithfully reported endogenous Olig2 expression in tumor. (B) Xist mRNA was abundant in total RNA from tumors generated in female mice, but absent from the ribosome-bound fraction (SD bars shown). (TIF) [file pone.0046965.s001.tif]

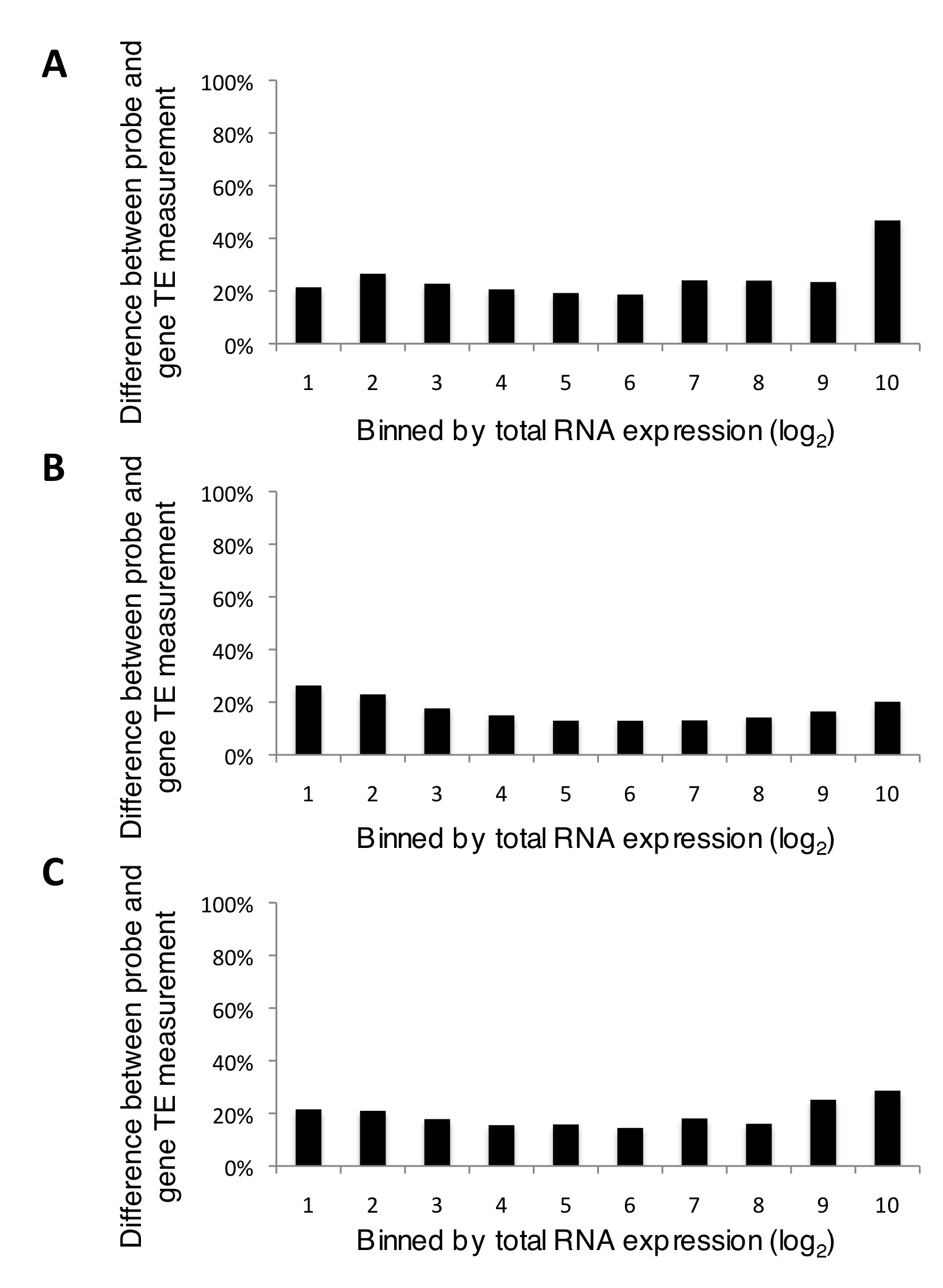

Supplement: Figure S2 — Multiple probes against the same gene identified similar TE measurements across the full range of expression values in total RNA. Average percent difference in measures TE between probe and gene average were binned by expression value in total RNA for (A) PDGF-driven mouse glioma, (B) normal brain OPCs and (C) PDGF-driven mouse glioma with PTEN deleted. (TIF) [file pone.0046965.s002.tif]

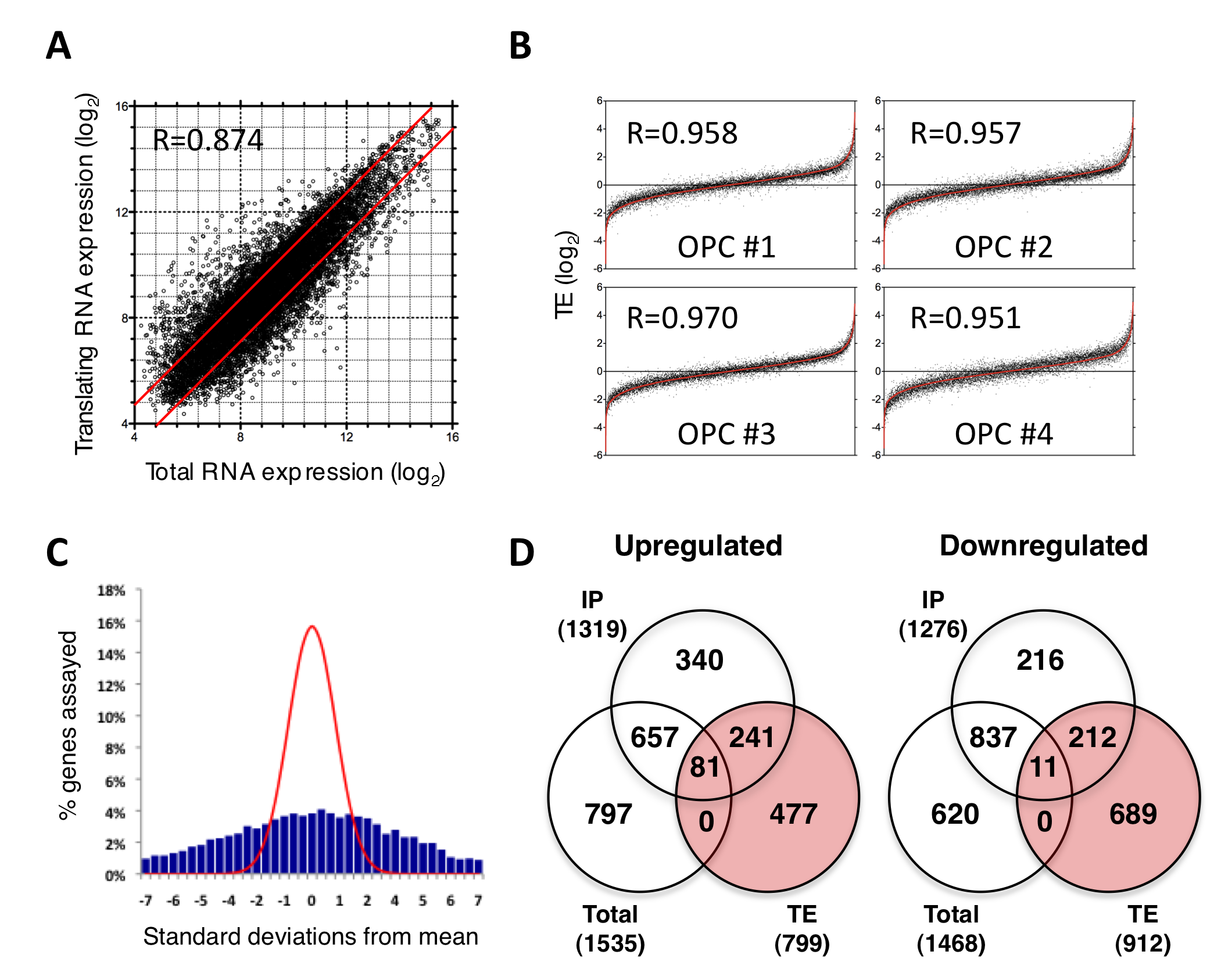

Supplement: Figure S3 — TE measurements in normal brain OPCs. (A) Distribution of mRNA expression in ribosome-bound and total RNA pools from normal brain OPCs identified differential ribosome recruitment efficiencies. (B) TE values for each biological replicate (black points) plotted with the average of the other three replicates (red line) demonstrated reproducibility of measurements. (C) Signal to noise ratios of TE measurements (blue bars) identified range of high confidence measurements relative to normal distribution (red line). (D) Venn diagram of probes changed greater than 2-fold (and FDR<0.05) between tumor and normal Olig2+cells in ribosome-bound RNA (IP), total cellular RNA (Total) and TE. Pearson correlation coefficients (R) are represented. (TIF) [file pone.0046965.s003.tif]

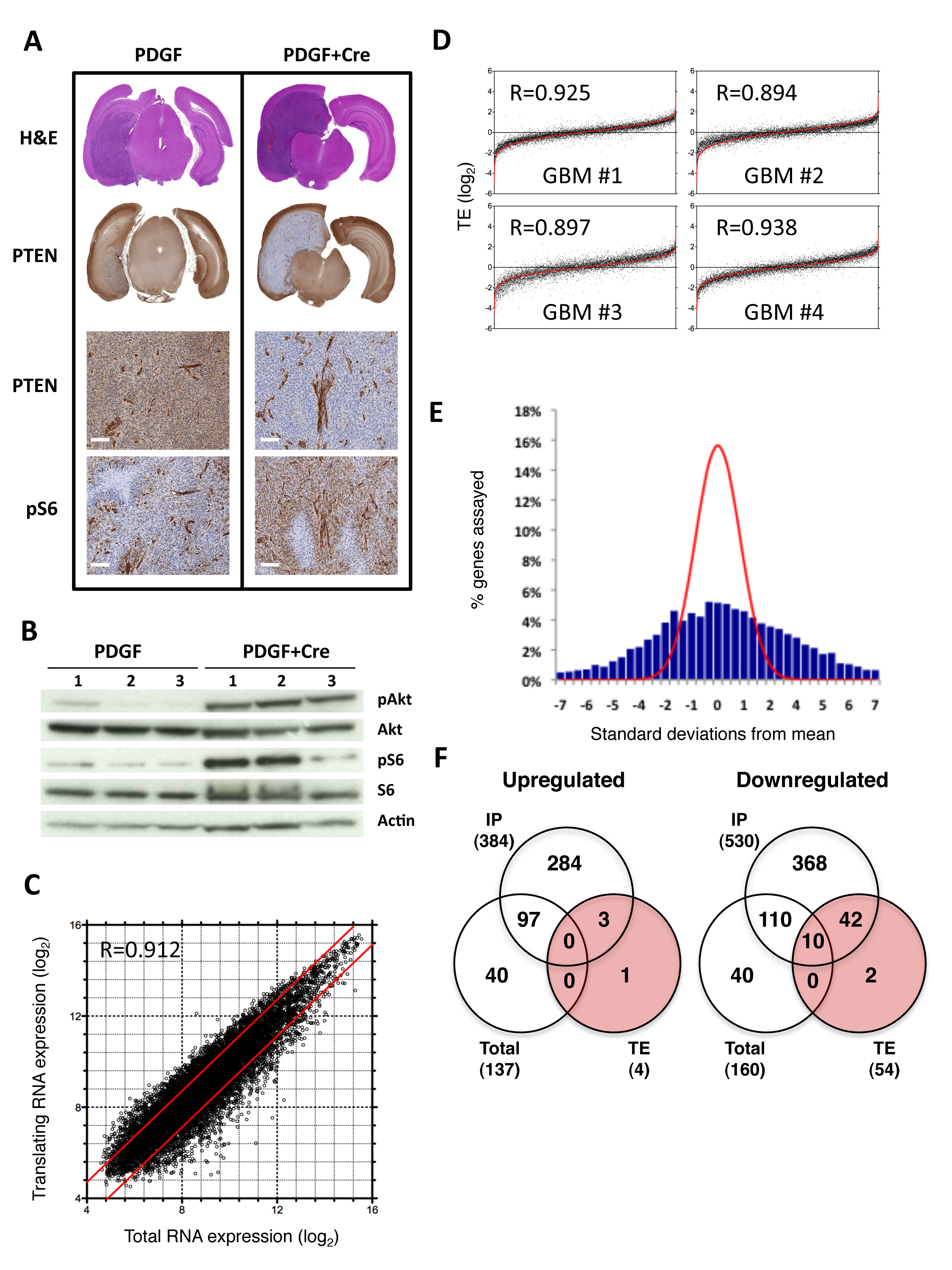

Supplement: Figure S4 — TE measurements in PDGF-driven glioma with PTEN deleted. (A) Immunohistochemical staining of tumor sections demonstrated the absence of PTEN protein and increased p-S6 positivity in mice receiving Cre virus in addition to PDGF. (B) Western blot of tumor lysates from mouse glioma demonstrates increased phosphorylation of Akt (T308) and S6 (S235/236) in tumors with PTEN deleted compared to tumors expressing PTEN. (C) Distribution of mRNA expression in ribosome-bound and total RNA pools from PDGF+Cre tumors identified differential translational efficiencies. (D) TE values for each biological replicate (black points) plotted with the average of the other three replicates (red line) demonstrated reproducibility of measurements. (E) Signal to noise ratios of TE measurements (blue bars) identified range of high confidence measurements relative to normal distribution (red line). (F) Venn diagram of probes changed greater than 2-fold (and FDR<0.05) between PTENfl/fl and PTEN−/− Olig2+cells in ribosome-bound RNA (IP), total cellular RNA (Total) and TE. (G) mRNAs involved in cellular respiration and encoding components of the electron transport chain were translationally downregulated upon PTEN loss. Pearson correlation coefficients (R) are represented. (TIF) [file pone.0046965.s004.tif]

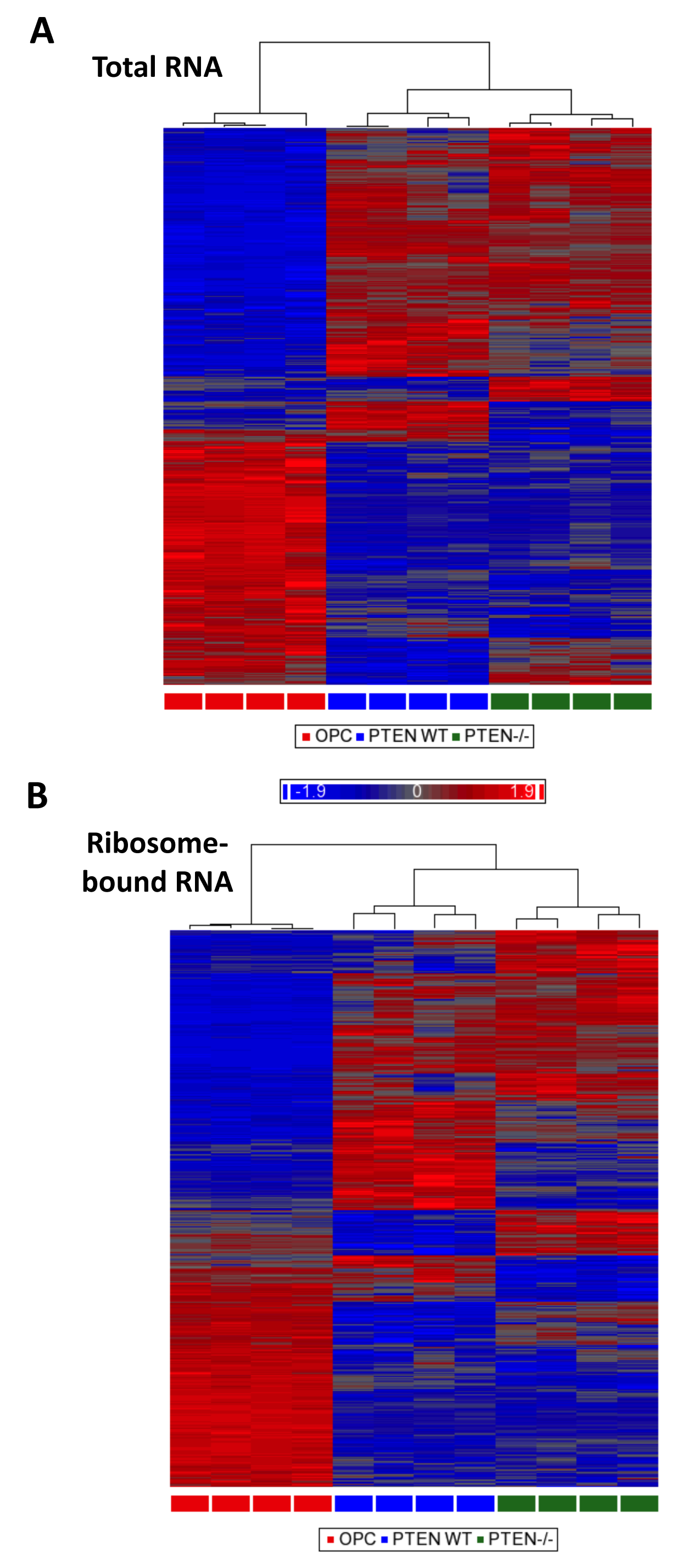

Supplement: Figure S5 — Hierachical clustering of normalized microarray expression data from (A) total cellular RNA and (B) ribosome-bound RNA extracted from Olig2+cells in normal brain and PDGF-driven glioma demonstrates the high degree of similarity of PTEN-expressing and PTEN-deleted tumors compared to normal brain OPCs. Hierachical clustering performed on transcripts significantly different by ANOVA at 0.05 FDR. (TIF) [file pone.0046965.s005.tif]
